# Correlations between subjective treatment responses and plantar pressure parameters of metatarsal pad treatment in metatarsalgia patients: a prospective study

**DOI:** 10.1186/1471-2474-7-95

**Published:** 2006-12-05

**Authors:** Jiunn-Horng Kang, Min-Der Chen, Shih-Ching Chen, Wei-Li Hsi

**Affiliations:** 1 Department of Physical Medicine and Rehabilitation, Taipei Medical University Hospital, 252 Wu-Xing Street, Taipei, Taiwan, R.O.C; 2 Department of Physical Medicine and Rehabilitation, Min-Sheng General Hospital, 168 Ching-Kuo Road, Taoyuan, Taiwan, R.O.C; 3 Department of Physical Medicine and Rehabilitation, National Taiwan University Hospital, 7 Chung Shan South Road, Taipei, Taiwan, R.O.C

## Abstract

**Background:**

Metatarsalgia is related to repetitive high-pressure loading under the metatarsal head (MH) that causes pain. The high pressure under the MH can be reduced by adequately applying metatarsal pads (MPs). Plantar pressure measurements may provide a method to objectively evaluate pressure loading under the MH. However, it is still unclear if the decrease in plantar pressure under the MH after MP treatment is associated with subjective improvement. This study aims to explore the correlations between subjective pain improvement and outcome rating, and the plantar pressure parameters in metatarsalgia patients treated using MPs.

**Methods:**

Thirteen patients (a total of 18 feet) with secondary metatarsalgia were included in this study. Teardrop-shaped MPs made of polyurethane foam were applied just proximal to the second MH by an experienced physiatrist. Insole plantar pressure was measured under the second MH before and after MP application. Visual analog scale (VAS) scores of pain were obtained from all subjects before and after 2 weeks of MP treatment. The subjects rated using four-point subjective outcome scales. The Wilcoxon signed-rank test was used to analyze the difference between the plantar pressure parameters and VAS scores before and after treatment. The Kruskal-Wallis test was applied to compare the plantar pressure parameters in each outcome group. Pearson's correlation was applied to analyze the correlation between the changes in plantar pressure parameters and VAS scores. Statistical significance was set as p < 0.05.

**Results:**

MP application decreased the maximal peak pressure (MPP) and pressure-time integral (PTI) under the second MH and also statistically improved subjective pain scores. However, neither the pre-treatment values of the MPP and PTI shift in the position of the MPP after treatment, nor the age, gender and body mass index (BMI) of the subjects were statistically correlated with subjective improvement. Declines in the PTI and MPP values after MP application were statistically correlated with the improvement in VAS scores (*r *= 0.77, *R*^2 ^= 0.59, *p *< 0.001; *r *= 0.60, *R*^2 ^= 0.36, *p *= 0.009).

**Conclusion:**

We found that the successful decline in the PTI and MPP under the second MH after MP application was correlated to subjective pain improvement. This study provides a strategy for the further design and application of MPs for metatarsalgia treatment.

## Background

Metatarsalgia is either a primary or secondary condition that causes great discomfort in daily living [1-4]. Primary metatarsalgia is mainly related to repetitive pressure loading under the MH, which exceeds the focal tissue tolerance and leads to inflammation and pain [1,2,5]. Many conditions such as pes cavus, pes planus, hyperpronated feet, hallux valgus and claw/hammer toe will increase pressure loading under the MH. In another way, fat pad atrophy, properties change (in DM or RA) or displacement (in claw/hammer toes) will decrease the tolerance of the tissue under the MH to the pressure loading. This disequilibrium in biomechanics is associated with metatarsalgia [2,3]. Secondary metatarsalgia has been associated with other conditions such as rheumatoid arthritis, ankylosing spondylitis, gout, diabetic mellitus, nerve entrapment, etc [1-3]. Besides pressure loading under the MH, the pathomechanism that includes an abnormal inflammation process, neuropathy, fat pad atrophy, etc. is more complex in secondary metatarsalgia.

Metatarsal pads (MPs) are placed just proximal to the MH and can redistribute the plantar pressure and transfer pressure loading under the MH to an adjacent area [6-8]. Using MPs is a useful, relatively less expensive and easily-applied method to treat metatarsalgia [9]. However, pressure under the MH may fail to reduce due to inadequate thickness or malpositioning of the MPs. Appropriate placement and size of the MPs are important to successfully relieve pressure under the MH [6-8,10]. The materials of the foot insert or MPs is another important issue. The mechanical properties of the materials including the abilities of force-distribution, shock absorption and durability should be considered thoroughly to achieve the maximal therapeutic effect [11,12]. In the standard laboratory testing, polyurethane foams are promising materials in the fabrication of foot orthotic [11]. Additional advantages of using these materials are comfortable, inexpensive, and easy to modify.

Plantar pressure measurements can monitor changes in plantar pressure before and after the MP application, which can provide a useful guide for appropriate MP placement [6-8,10]. Hayda et al. and Holmes et al. showed that MP placement can effectively reduce pressure under the MH in healthy subjects [6,7]. Our previous study demonstrated the distribution pattern of plantar pressure under the MH in healthy and metatarsalgia subjects. MP placement was also effective in metatarsalgia patients [8,13].

Theoretically, the symptoms of metatarsalgia should improve after pressure under the MH is relieved by MPs. However, subjective treatment responses of metatarsalgia patients to MPs differed among clinical practices. The correlation between the subjective treatment responses and change in the plantar pressure parameters after MP placement in metatarsalgia patients remained unclear. We hypothesize that poor subjective improvement is related to the failure to reduce plantar pressure under the MH using MPs. Our study aims to obtain plantar pressure parameters by measuring the insole plantar pressure before and after MP placement and analysing the correlation between subjective outcome ratings and pain improvement and the plantar pressure parameters under the MH.

## Methods

Taipei Medical University Hospital Review Board approved this study. Subjects with tenderness at the plantar surface or weight-bearing pain under the second MH for longer than 2 weeks that could not be relieved by medication were recruited at the clinic of the Department of Physical Medicine and Rehabilitation of Taipei Medical University Hospital from July 2004 to December 2004. After an initial examination by an experienced physiatrist, patients with conditions such as fixed feet deformity, severe feet malalignment (pes cavus, pes planus, hyperpronated feet, hallux valgus and claw/hammer toes), previous feet surgery or major trauma, diabetic mellitus, ankylosing spondylitis, rheumatoid arthritis and nerve entrapments (Morton's neuroma) were excluded. Thirteen patients (a total of 18 feet) were included in this study. Signed, informed consent was obtained from all subjects.

The visual analog scale (VAS), a horizontal line 10 cm in length, was self-scored. The word anchored at the left end was 'no pain' and at the right end was 'almost intolerable pain'. Patients were asked to mark the point corresponding to their average pain level when walking. The VAS was measured in centimetres using a ruler, and a score was recorded (0.0–10.0). VAS scores for pain were recorded for all subjects before MP placement.

The patients' shoes were checked first. Replacement with new or other shoes was suggested if the condition of their original shoes was not suitable for MP placement, such as the heels being too high, toe-box too narrow, or shoes too worn out. The only premise of the study was that the subjects use suitable shoes. Insole plantar pressure was measured using Pliance 16P (Novel GmbH, Munich, Germany), a stretchable high-resolution capacitive sensor containing 256 sensors of 2.8 × 2.8 mm and forming a 16 × 16 matrix. The pressure sensor matrix was taped to the plantar surface under the second MH region of a subject's feet. Subjects performed at least three walking trials prior to formal testing to check for any inadequacy in the sensor positions or evident gliding between interfaces and to determine their average most comfortable walking speed. Plantar pressure data were recorded when subjects walked at their most comfortable speed along a 10-m walkway wearing their shoes for a total of six trials. We did not control the subjects' walking speed by any audio or visual signals; instead the subjects were requested to walk at their most comfortable speed. If a subject's walking speed deviated form his/her average most comfortable walking speed by more than 15% in the formal test, it was abandoned. Pressure data were recorded at a sampling frequency of 38 Hz. After the above mentioned plantar pressure measurement was completed, a 55 × 40 × 8 mm teardrop-shaped MP made of polyurethane foam (Schein Orthopadia Service, Remscheid, Germany) was fixed to the insole just proximal to the second MH by an experienced physiatrist. An identical measurement protocol was followed after MP placement.

The first and terminal steps of a row of data of each trial were removed before data analysis to exclude the effects of acceleration and deceleration. Peak pressure was defined as the highest pressure in each sensor in the matrix occurring in one step (from forefoot loading to push off), and the maximal peak pressure (MPP) was identified as the highest-pressure sensor of peak pressure under the second MH area in the matrix. The PTI was defined as the area under the pressure-time curve of each step at the sensor where the MPP occurred. Two representative steps were selected in 1 trial and a total 12 steps in a subject was selected for further data averaging (2 steps × 6 trials). The pre- and post-treatment positions where the MPP occurred in the matrix were also recorded, and the distance of the shift (DS) in MPP position was calculated.

After MP placement and plantar pressure measurement, the subjects were requested to wear their shoes with the newly padded insole when walking. All medications for analgesic purposes were discontinued. Patients were followed up in the outpatient clinic after 2 weeks. The subjective overall treatment response was reported as the following 4 categories: (1) worse, (2) no change, (3) mild improvement with a subjective improvement of <50% and (4) major improvement with a subjective improvement of >50%. VAS scores during normal walking were recorded for all the subjects at follow-up.

The Wilcoxon signed-rank test was applied to compare paired parameters of plantar pressure data and the VAS score changes before and after MP application. The Kruskal-Wallis test was applied to compare the plantar pressure parameters and basic variables among in each outcome group. Scheffe's test was applied for the host-hoc test. Pearson's correlation test was applied to analyze the correlation between the plantar pressure parameters and change in the VAS scores after treatment. The alpha value was set to 0.05.

## Results

### Basic profiles of subjects

A total of 13 subjects were included in this study. There were 9 females and 4 males aged 50.5 ± 13.1 (range, 28 to 67) years. The symptoms involved both feet in 5 patients. There were a total of 18 feet with symptoms of secondary metatarsalgia – 7 on the right side and 11 on the left side. Body weight, height, and body mass index (BMI) of the subjects were 61.6 ± 11.6 kg, 161.7 ± 9.7 cm and 23.0 ± 3.5 kg/m^2^, respectively. The duration of the metatarsalgia symptoms ranged from 2 weeks to 1 year. All the basic variables of the subjects are listed in Table 1.

### Plantar pressure profiles

Plantar pressure was measured in all the subjects, and the results are listed in Table 2. The pre-treatment MPP of all the subjects was 225.8 (95% CI, 174.5–280.0) kPa, and the PTI was 34.8 (95% CI, 27.0–42.6) kPa·s. After MP placement, the MPP and PTI of all the subjects decreased significantly; their values were 199.0 (95% CI, 163.3–234.8) kPa and 31.9 (95% CI, 24.3–39.5) kPa·s, respectively.

**Figure 1:**
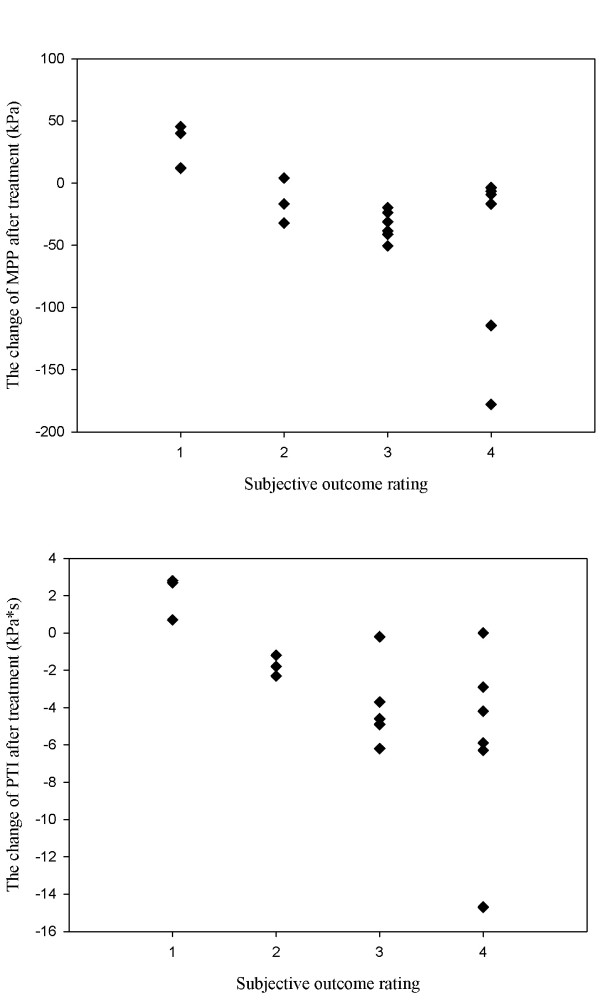
**Changes in the PTI and MPP values after MP placement in each outcome group**. The difference between two parameters differed significantly in each outcome group (*p *< 0.05). Note that the MPP of 4 feet increased after MP placement–3 rated the outcome as 1, while one rated it as no change, i.e. Subjective outcome rating: 1, worse; 2, no change; 3, improvement <50%; 4, improvement >50%. MPP, maximal peak pressure; PTI, pressure time integral.

The mean change in the MPP was -26.7 (95% CI, -52.3 to -1.1) kPa, and the PTI was -2.9 (95% CI, -5.0 to -0.9) kPa·s (Table 2). However, we still found 4 feet in which the MPP was elevated after MP placement (Figure 1). The position of MPP occurrence in the matrix shifted significantly after treatment, and the mean distance of shifting was 3.6 (95% CI, 2.5 to 4.5) mm.

### Subjective treatment responses

**Figure 2:**
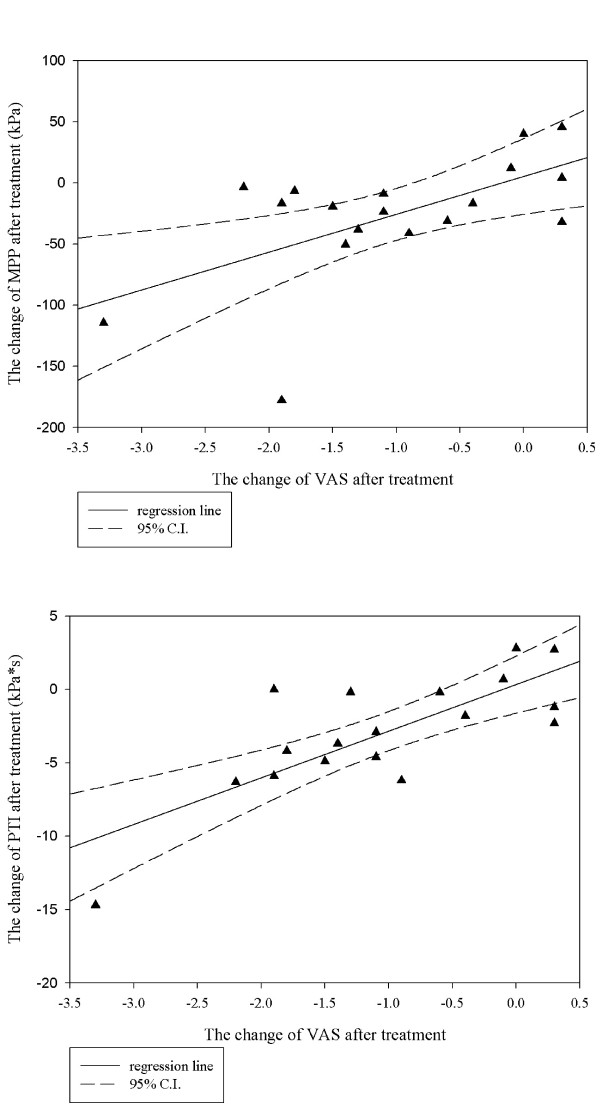
**The scatter graph with regression line (95% CI) of the changes in the PTI and MPP versus changes in the VAS score after MP placement**. The correlations of the changes in VAS scores with changes in the MPP and PTI values were statistically significant (*r *= 0.77, *R*^2 ^= 0.59, *p *< 0.001; *r *= 0.60, *R*^2 ^= 0.36, *p *< 0.009). MPP, maximal peak pressure; PTI, pressure time integral; VAS, visual analog scale. CI, confidence interval.

None of the subjects dropped out or discarded their newly padded insole shoes in the follow-up. The VAS scores during normal walking decreased significantly after MP placement. The mean change in the VAS scores was -1.0 (95% CI, -1.5 to -0.5). The patient's subjective outcome rating at follow-up rated 3 feet as 'worse', 3 as 'no change', 6 as 'mild improvement' and 6 as 'major improvement'. The plantar pressure parameters for each outcome group are listed in Table 3. We found that the decline in the MPP and PTI after MP placement differed significantly in each outcome group (*p *= 0.033 and *p *= 0.034, respectively). The patient's age, gender or symptom duration did not differ significantly within an outcome group. Declines in the PTI and MPP values after MP placement significantly correlated to the decrease in the VAS scores (*r *= 0.77, *R*^2 ^= 0.59, *p *< 0.001; *r *= 0.60, *R*^2 ^= 0.36, *p *< 0.009) (Figure 2). The pre- and post-treatment MPP and PTI values or the DS in the MPP position in the matrix were not statistically correlated to changes in the VAS (Table 4).

## Discussion

In previous studies, both barefoot and insole plantar pressure measurements were conducted to monitor plantar pressure [5,14-16]. However, the barefoot measurement may not reflect the actual biomechanical effect after MP placement in the daily life, since the interaction among MPs, feet and shoes cannot be studied. To study the effects of MPs within the shoe environment, Chang et al. conducted a multi-step insole protocol to demonstrate the pressure redistribution effects of MPs in healthy subjects [15]. We also used a multi-step protocol with insole plantar pressure measurement to simulate the daily walking activity of the subjects with original and newly padded shoes as closely as possible. We believe this protocol can provide more accurate estimates of the plantar pressure parameters than barefoot or single-step protocols.

Previously, we demonstrated that optimal positioning of the MPs reduces 28% of peak pressure under the MH, and this position was at just proximal to the second MH using barefoot measurement [8]. We applied this finding to our current research but found an average decline of only 11.8% in the MPP value by insole measurement; this was much less than the decline in the MPP value observed in our previous data. We believe that this discrepancy is because of the biomechanical interaction among the shoes, MPs and feet when using insole measurement. Hayda et al. suggested that positioning the MP at 5 mm distal to the MH causes the greatest reduction in plantar pressure; in addition, they found that plantar pressure failed to decline in 40% of the male feet in his study; this was 22% in our study [7]. These conflicting findings may also be a reflection of the considerable individual anatomical and biomechanical variations that influence the optimal positioning of MPs. Moreover, using an external body marker to place the MPs is not a reliable method to reduce pressure under the MH. In conclusion, we suggest that plantar pressure measurement can be used as an objective tool to guide MP placement. Further, we suggest that the optimal position of the MPs should be individualized by using the patients' own shoe. Replacement or changing to different-sized MPs should be considered when MP placement fails to achieve PTI and MPP reduction by insole plantar pressure measurement.

We found that the declines in the PTI and MPP under the MH after MP application correlated with significant pain improvement. The increase in the MPP of 4 feet after MP placement is also noteworthy; of these, 3 rated the symptoms as worse, while one rated as no improvement during the follow-up. This finding demonstrates that subjects in whom MP placement failed to reduce pressure under the MH may experience no subjective improvement. However, Postema et al. showed that the reduction in peak plantar pressure was not correlated to pain score improvement in metatarsalgia patients treated by a rocker bar [5]. Even the biomechanical effects of the rocker bar and MPs differed; the former decreased the inclination of the metatarsus, while the latter redistributed the pressure to an adjacent tissue. However, both foot orthotic were beneficial in reducing pressure under the MH. We believe that these inconsistent results may be related to the pain modulation effect of the MPs. Since the MPs reduced pressure under the MH by transferring pressure loading to the adjacent tissues, this phenomenon may increase the sensory input of the deep pressure (large-fibre afferents) of the adjacent tissue; metatarsalgia could be modulated according to the 'gate-control theory' [17,18]. Hodge et al. found only 32% variance in pain was determined by measuring the average pressure under the MH in metatarsalgia due to rheumatoid arthritis, and he concluded that the pain sensation may play a role in this observation [14]. Similarly, Postema et al. noted that a subject with pain preferred to use a custom moulded insole over a rocker bar, although both orthrosis reduce plantar pressure [5]. We believe that the pain modulation effect of MPs may be one of mechanisms of pain relief in metatarsalgia. Further study is needed to examine this hypothesis.

We found that the decline in the PTI value after MP placement correlated well with that in the VAS scores, while the decline in the MPP value correlated moderately (*r *= 0.77, *R*^2 ^= 0.59 versus *r *= 0.60, *R*^2 ^= 0.36). There are several possible explanations for this finding. First, considering a temporal effect, the PTI provides a better estimation of overall pressure loading under the MH than the MPP, which is a point estimation. Second, nociceptors have slower responses to increasing pressure than mechanoreceptors [19]. The brief duration of the peak pressure under the MH may be insufficient to cause high frequency firing of the nociceptors [14,19]. Based on our findings, we suggest that the optimal treatment should be lowering the PTI under the MH as much as possible.

We found that the MPP and PTI values before and after treatment did not correlate to the subjective outcome rating or pain improvement. Previous studies showed that plantar pressure of asymptomatic subjects exhibit large variation [6,13,14]. The normal thresholds of plantar pressure under a human MH that induce tissue damage are difficult to determine. Hence, it is difficult to set treatment goals for lowering the PTI or MPP to a constant threshold. Although a shift in the position of the peak pressure may be observed after MP application, we found that the distance by which the peak pressure shifted after treatment was not correlated to the subjective outcomes or declines in the VAS scores. We concluded that it is neither to shift the 'peak pressure site' from the painful metatarsal area as far as possible nor lower the PTI and MPP to a 'normal threshold' to achieve a good treatment result.

There were some insufficiencies in our study. First, we studied a small number of cases. The statistical power of our study was 0.25–0.33 at a sample size of 18, and the alpha level set as 0.05. The type II error should be considered when the statements of the differences between variables were insignificant. Second, to ensure homogeneity of the subjects, we excluded conditions such as severe hyperpronated feet, pes cavus, claw/hammer toes, and hallux valgus, which are not uncommon in clinical practice. The biomechanics of these conditions are various and may require different orthrosis considerations [3,9]. Third, analysis of the data obtained by combining that from both feet of the same person as in the case of some subjects may violate the assumption of independence of the data set. There is still no data available to analyze the difference between the characteristics of unilateral and bilateral metatarsalgia patients. The statistical methods used to analyze the correlated dataset in the treatment response of patients who had bilateral metatarsalgia should be more adequate. Finally, our subjects were followed up after 2 weeks. Follow-up for a longer period may be needed to observe long-term outcomes.

Our findings have several implications. First, using real-time plantar pressure measurements before and after MP placement can provide a tool to monitor treatment in clinical practice. Readjustment of MP placements for those who failed to demonstrate a decline in the PTI and MPP values after treatment should be considered to improve treatment results. Second, the PTI can estimate overall pressure-loading of the tissue under the MH and may be a more important marker than the MPP. This finding can be applied to a new insert or insole design. Finally, the absolute values of the MPP and PTI are not correlated to the treatment results. Successful lowering of the MPP and PTI is key for symptom relief.

## Conclusion

Applying MPs is an effective method to reduce pressure loading under the MH and relieve the symptoms of metatarsalgia. We found that the decline in the PTI and MPP values after MP application was correlated to subjective pain improvement. We suggest that MP placement for patients with metatarsalgia should be individualized and adjusted by the monitoring of the plantar pressure measurements. This study can also provide a strategy for further orthrosis design and may be used for metatarsalgia treatment.

## Competing interests

The author(s) declare that they have no competing interests.

## Authors' contributions

JHK, MDC and WLH were responsible for the study design. JHK and CSC recruited the clinical subjects. JHK and WLH conducted the plantar pressure measurements and data analysis. MDC and CSC contributed to the conceptual framework of the study. All authors read and approved the final manuscript.

## Pre-publication history

The pre-publication history for this paper can be accessed here:



## Figures and Tables

**Table 1:** Basic variables of subjects

	Total (mean ± SD)	Range
Age (years)	50.5 ± 13.1	28 ~ 67
BMI (kg/m^2^)	23.0 ± 3.5	18.3 ~ 28.9
Symptom duration (weeks)	18.9 ± 17.0	2 ~ 52

Gender (F:M)	9:4	
Involved side (right: left)	7:11	

BMI, body mass index.

**Table 2:** Plantar pressure parameters and VAS (mean and 95% CI) before and after MP placement

	Pre-treatment	Post-treatment	Change after treatment	*p *value
MPP (kPa)	225.8 (174.5 ~ 280.0)	199.0 (163.3 ~ 234.8)	-26.7 (-52.3 ~ -1.1)	0.028
PTI (kPa·s)	34.8 (27.0 ~ 42.6)	31.9 (24.3 ~ 39.5)	-2.9 (-5.0 ~ -0.9)	0.005
VAS	4.9 (4.4 ~ 5.5)	3.9 (3.1 ~ 4.7)	-1.0 (-1.5 ~ -0.5)	0.001

MPP, maximal peak pressure; PTI, pressure time integral; VAS, visual analog scale; CI, confidence interval.

**Table 3:** Plantar pressure parameters (mean and 95% CI) in each subjective outcome group

Subjective outcome	1 (*n *= 3)	2 (*n *= 3)	3 (*n *= 6)	4 (*n *= 6)	*p*
pre-MPP (kPa)	175.7 (132.0 ~ 219.4)	190.9 (-30.4 ~ 412.2)	215.5 (167.8 ~ 263.2)	278.5 (278.4 ~ 444.5)	0.621
post-MPP	208.2 (120.8 ~ 295.6)	176.0 (-5.9 ~ 357.8)	181.4 (144.2 ~ 218.6)	223.7 (107.7 ~ 339.7)	0.683
MPP-change	32.5 (-11.9 ~ 96.9)	-14.9 (-60.0 ~ 30.2)	-34.1 (-46.2 ~ -22.0)	-54.8 (-132.1 ~ 22.6)	0.033

pre-PTI (kPa·s)	26.0 (17.4 ~ 34.6)	37.7 (-5.6 ~ 81.0)	30.7 (24.1 ~ 37.4)	41.9 (17.5 ~ 66.3)	0.730
post-PTI	2.80 (18.4 ~ 37.6)	35.9 (-7.8 ~ 79.7)	27.4 (21.3 ~ 33.6)	36.3 (11.4 ~ 61.1)	0.981
PTI-change	2.1 (-0.9 ~ 5.0)	-1.8 (-3.1 ~ -0.4)	-3.3 (-6.0 ~ -0.6)	-5.7 (-10.9 ~ -4.4)	0.034

DS of MPP (mm)	5.8 (4.0 ~ 6.0)	2.9 (0 ~ 8.8)	2.7 (1.5 ~ 4.0)	3.0 (1.2 ~ 5.0)	0.086

pre, pre-treatment; post, post-treatment; change, post-treatment value – pre-treatment value; DS, distance of shifting; CI, confidence interval. Subjective outcome rating: 1, worse; 2, no change; 3, improvement <50%; 4, improvement >50%.

**Table 4:** Pearson's correlation test between the change of VAS and the plantar pressure parameters

Correlation with VAS-change	*r*(*R*^2^)	*p*-value
pre-MPP	-0.447 (0.20)	0.063
post-MPP	-0.21 (0.04)	0.402
MPP-change	0.60 (0.36)	0.009

pre-PTI	-0.292 (0.09)	0.240
post-PTI	-0.093 (0.008)	0.714
PTI-change	0.77 (0.59)	<0.001

DS of MPP	0.01 (<0.001)	0.969

pre, pre-treatment; post, post-treatment; change, post-treatment value – pre-treatment value; DS, distance of shifting.
